# Amino acid motifs for the identification of novel protein interactants

**DOI:** 10.1016/j.csbj.2022.12.012

**Published:** 2022-12-10

**Authors:** Aloysius Wong, Chuyun Bi, Wei Chi, Ningxin Hu, Chris Gehring

**Affiliations:** a Department of Biology, College of Science and Technology, Wenzhou-Kean University, 88 Daxue Road, Ouhai, Wenzhou, Zhejiang Province 325060, China; b Wenzhou Municipal Key Lab for Applied Biomedical and Biopharmaceutical Informatics, Ouhai, Wenzhou, Zhejiang Province 325060, China; c Zhejiang Bioinformatics International Science and Technology Cooperation Center, Ouhai, Wenzhou, Zhejiang Province 325060, China; d Department of Chemistry, Biology & Biotechnology, University of Perugia, Perugia 06121, Italy

**Keywords:** Amino acid motifs, Moonlighting proteins, Functional centers, Search motif, Hidden domains, Gas sensing, Hormone perception, Sequence analysis, Protein interactants

## Abstract

Biological systems consist of multiple components of different physical and chemical properties that require complex and dynamic regulatory loops to function efficiently. The discovery of ever more novel interacting sites in complex proteins suggests that we are only beginning to understand how cellular and biological functions are integrated and tuned at the molecular and systems levels. Here we review recently discovered interacting sites which have been identified through rationally designed amino acid motifs diagnostic for specific molecular functions, including enzymatic activities and ligand-binding properties. We specifically discuss the nature of the latter using as examples, novel hormone recognition and gas sensing sites that occur in moonlighting protein complexes. Drawing evidence from the current literature, we discuss the potential implications at the cellular, tissue, and/or organismal levels of such non-catalytic interacting sites and provide several promising avenues for the expansion of amino acid motif searches to discover hitherto unknown protein interactants and interaction networks. We believe this knowledge will unearth unexpected functions in both new and well-characterized proteins, thus filling existing conceptual gaps or opening new avenues for applications either as drug targets or tools in pharmacology, cell biology and bio-catalysis. Beyond this, motif searches may also support the design of novel, effective and sustainable approaches to crop improvements and the development of new therapeutics.

## 1 Introduction

**Fig. 1:**
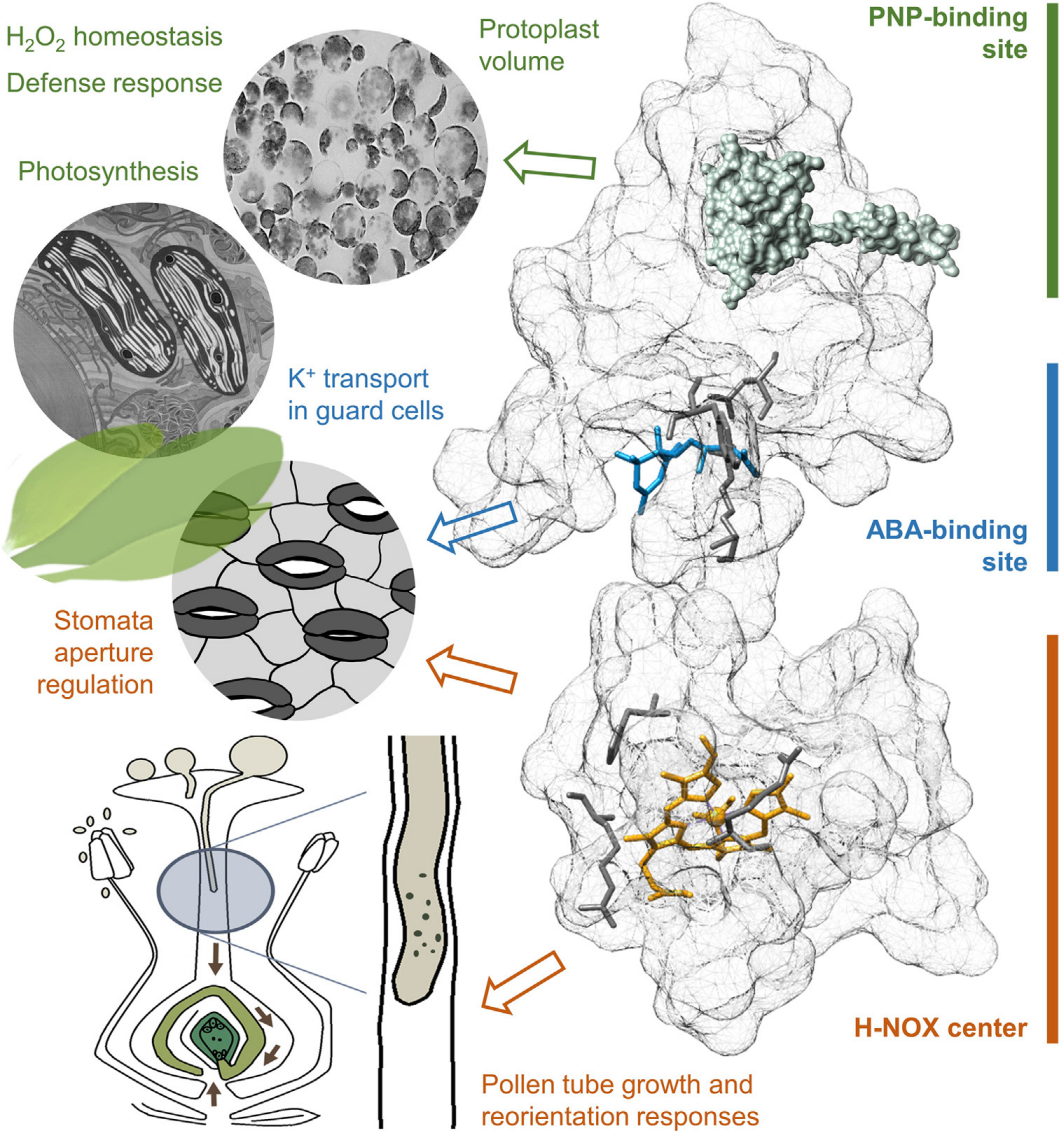
**The physiological functions of non-enzymatic protein interacting sites identified through amino acid motifs.** The hypothetical protein model shows the different interacting sites docked with their respective ligands. The PNP-binding site (green), the ABA-binding site (blue), and the H-NOX center (orange) are represented, with the key amino acids involved in interactions with their ligands shown in dark grey. The PNP-binding site is implicated in H_2_O_2_ homeostasis, regulation of protoplast volume, defense response against pathogens, and photosynthesis, which could be achieved through interactions of PNP-A (TAIR: At2g18660) with PNP-receptor 1 (TAIR: At1g33612), catalase 2 (TAIR: At4g35090) or Rubisco activase (TAIR: At2g39730) [106], [107], [108], [109], [110]. The ABA-binding site of an outward rectifying potassium channel AtGORK (TAIR: At5g37500) is involved in K^+^ transport in guard cells [95]. The H-NOX center of a pollen-specific DIACYLGLYCEROL kinase AtDGK4 (TAIR: At5g57690) [138] has been shown to affect NO-mediated pollen tube growth and reorientation response while the H-NOX center of AtNOGC1 is implicated in ABA-mediated stomata closure [134], [135]. The protein model and molecular graphics were constructed and prepared using UCSF Chimera [172] and molecular docking was performed using AutoDock Vina [173]. Chloroplast and protoplast images were obtained from the Wellcome Library (https://wellcomelibrary.org) and Wikimedia Commons (https://commons.wikimedia.org). Other images were created with Procreate for iPad, and Microsoft PowerPoint.

Amino acid motifs based only on conserved amino acids with critical biochemical roles can be constructed to enhance the discovery of novel protein interactants [1], [2]. Such interactions include ligands such as hormones, second messengers, gases, or indeed other proteins. Conserved amino acid motifs have already proved successful in the discovery of signaling components in plants many of which have no orthologues or sequence homology with proteins in other organisms including animals, fungi, other eukaryotes, and bacteria [3], [4], [5], [6]. One likely reason for this is that plant proteins have evolved complex and plant specific domain architectures which put for example, moonlighting catalytic centers beyond the detection limits of alignment tools such as BLAST. As complex proteins with multiple functions, they can enable highly precise, rapid, and tailored regulations of metabolic and cellular processes thereby increasing the plasticity of responses to environmental stresses, both biotic and abiotic in nature [7], [8], [9], [10], [11], [12]. Such an ability is crucial and possibly particularly pertinent to sessile organisms that lack the ability to physically evade the source of the stress. The lack of physical mobility necessitates complex adaptive responses at the organismal level as well as metabolic changes at the cellular level [13], [14], [15], [16], [17], [18], [19], [20], [21], [22], [23], [24].Fig. 1.

Carefully curated amino acid motifs were first applied for the identification of proteins with enzymatic activities such as guanylate cyclases (GCs) and adenylate cyclases (ACs) many of which, reside within larger domains such as kinases, and often constituting only a small region of a much larger multidomain protein [25], [26], [27], [28], [29], [30], [31], [32], [33]. These complex proteins with AC or GC activities perform primary functions including acting as transporters, enzymes, and receptors or they participate in protein-protein interactions and binding to other ligands. Identification of GCs and ACs that moonlight within complex proteins have in recent years been extended to crop plants such as tomato, maize, and apple, as well as to other systems such as the discovery of a GC in a human interleukin-1 receptor-associated kinase-3 (IRAK3) which is a critical checkpoint molecule for inflammatory responses [33], [34], [35], [36], [37], [38], [39], [40]. Additionally, the degrading enzymes for cyclic nucleotides, the phosphodiesterases (PDEs), have also recently been identified in both monocots and dicots using an analogous motif search. These searches have revealed interesting novel twin domain architectures where both cyclase and PDE domains occur within the same protein conceivably enabling dynamic and intricate tuning of cyclic mononucleotide signal strengths and concomitantly also, their downstream effects [41], [42], [43]. These twin domain proteins therefore serve as attractive targets for biotechnological innovations aiming at crop improvements [44], [45].

Evidence for other moonlighting sites with for example ligand-binding properties, that were also identified with motif search approach, have recently emerged. Here, we provide a focused review on the nature of such molecules discovered to-date and propose potential implications for their effects at the cell, tissue, or organismal level. We also discuss several promising avenues for the expansion of amino acid motif searches to discover hitherto unknown protein interactants and interaction networks.

## 2 Hormone perception

Abscisic acid (ABA) is a well-established phytohormone that is critical for plant growth, development, and defense [46], [47]. Importantly, as a stress hormone, ABA integrates responses to environmental cues and orchestrates cellular events leading to appropriate physiological responses that confer different levels of tolerance to many biotic and abiotic stresses [48], [49]. Since the level of ABA normally increases in response to abiotic stresses, exogenous treatment of ABA has successfully increased crop tolerance to drought as it lowers transpiration rates through the modulation of stomatal aperture [50], [51]. ABA has also been applied to alleviate the harmful effects of heat and salt stresses and to improve grain yield and quality, among many other crop improvement initiatives [52], [53], [54]. Responses to ABA are enabled by the canonical ABA receptors PYR/PYL/RCAR and their complexes, and down-stream processes that link hormone perception to signaling pathways resulting in highly tuned stress responses in the shoots and the roots [55], [56], [57], [58]. Consequently, various approaches for crop improvements such as the genetic engineering of ABA signal transduction pathways, and the use of agrochemicals, have been attempted and, in parts, implemented [59], [60], [61].

Nevertheless, several studies have shown that the canonical ABA receptors cannot account for all plant processes and responses, indicating the presence of undiscovered ABA-responsive proteins [62], [63]. This notion is strengthened by the fact that the ABA, which is a conserved ancient signaling molecule that exist in algae, liverworts, fungus, and animal tissues, does not exert all its effects through the PYR/PYL/RCAR ABA receptors [64], [65], [66]. In mammals, ABA affects the innate immune response, stimulates mesenchymal and hemopoietic stem cell proliferation, and regulates cell glucose uptake and metabolism [67], [68], [69], [70], [71], [72], [73]. The mammalian lanthionine synthase C-like (LANCL) proteins LANCL1 and LANCL2, which share high structural homology with the LANC in bacteria, have been identified as *bona fide* ABA receptors [74], [75]. In bacteria, LANC catalyzes the transfer of a thiol from cysteine to a dehydrated serine which produces the lanthipeptides that have antimicrobial properties [76], [77]. Unlike the bacterial LANC, mammalian LANCL is not involved in lanthionine synthesis as knockdown of all LANCL isoforms do not reduce lanthionine levels in the brain of rats although ABA has already been shown to improve memory, learning and synaptogenesis through the NDR1/2 kinase pathway, thus raising the possibility of different molecular and physiological functions for mammalian LANCL [78], [79], [80]. Another possible role for mammalian LANCL is energy metabolism. In muscle cells for instance, LANCL1 has been shown to bind ABA and trigger glucose uptake [75]. Both LANCL1 and LANCL2 activate glucose transporters GLUT4 and GLUT1, and the signaling proteins in the AMPK/PGC-1α/Sirt1 pathway, stimulating respiration in mitochondria, while also protecting mitochondria of cardiomyocytes from hypoxia-induced injury through AMPK- and nitric oxide (NO)-mediated mechanisms [75], [81]. Previously, LANCL2 has already been shown to bind ABA on the membrane of human granulocytes which is necessary for ABA signal transduction in granulocytes and in insulinoma cells [74]. Structurally, LANCL2 is reminiscent of a typical peptide and steroid hormone receptor but it is not a transmembrane protein. LANCL2 is anchored through a myristoyl group at the intracellular side of the plasma membrane where it interacts with the α subunit of a G_i_ protein leading to the activation of AC, which then initiates the ABA signaling pathway [67], [71], [82]. Demyristoylation of LANCL2 results in translocation into the nucleus [83]. The downstream signaling of LANCL2 has also been elucidated where in response to insulin, ABA activates the AMPK/PGC-1α pathway or mTORC2 and interacts with PPAR-γ which in turns, activates adipogenic genes in white adipocytes [84], [85], [86]. ABA signaling through LANCL2 may also involve a pertussis toxin sensitive G protein that can activate an AC, whose product cAMP, then enables downstream signaling events including the phosphorylation of ADP-ribosyl cyclase CD38 by PKA, yielding cADPR and ADPR which eventually causes the release Ca^2+^ from intracellular stores [67], [71]. Identification of LANCL as ABA receptors in mammals enabled the development of synthetic ABA antagonists to reduce inflammation and to develop potentially new drugs for the treatment of diabetes [87], [88], [89], [90]. Interestingly, a recent study that employed an affinity-based method based on customized biotin linkers consisting of both alkyne and amino groups, a protein cross-linker, and ABA azido probes, has identified a cytosolic thioredoxin from *Arabidopsis thaliana* AtTrxh3 as an ABA-binding protein. Although the physiological effects of ABA-binding to AtTrxh3 have yet to be resolved, AtThrxh3 has already been implicated in many ABA-mediated responses e.g., to biotic stresses and elicitors such as fungal and microbial phytotoxin, and to abiotic stresses such as hydrogen peroxide and heat besides aiding protein folding and complex formation [91], [92], [93], [94]. This finding is consistent with the notion that there are ABA-binding proteins beyond the canonical PYR/PYL/RCAR that await discovery.

An ABA motif DX{7,8}RX{3,4}DX{5,6}YX{6,7}H was created based on key amino acids of the canonical ABA receptors and this motif identified an outward rectifying K^+^ channel in *Arabidopsis thaliana* guard cells (GORK; At5g37500) as an ABA-binding protein [95]. At the transcript level, GORK is expressed in response to various abiotic stresses and at the protein level and GORK can be modulated by a number of signaling molecules including cyclic nucleotides, ATP, lipids, phosphatases, G-proteins, GABA, and ABA, thus making it a ‘master switch’ of cellular metabolism [96]. Electrophysiological studies have revealed that the natural (±)-ABA but not the less active (-)-ABA isomer in the patch pipette increases the GORK current amplitude by 2.55-fold when expressed in HEK293 cells. Additionally, in the excised inside-out patch configuration where the cytosolic side of GORK is exposed to the bath solution and allowing the assessment of unitary single-channel recordings to be made before and after ABA application on the same excised membrane patch, the physiologically active (±)-ABA increased the opening probability of GORK by an average of 3.6-fold. This effect that is not observed with the (-)-ABA isomer. When two key amino acids at the ABA-binding site of GORK were mutated, the ABA-dependent GORK current amplitude was markedly reduced to levels almost comparable with the inactive (-)-ABA isomer. Consistently, a colorimetric based ELISA method developed to determine the affinity of ABA at the ABA-interacting site of GORK, yields a linear increase in signal in the presence of (±)-ABA but not with GORK harboring the same mutations [95]. Notably, the ABA-binding site of GORK is reminiscence of the latch-like region of the ABA-binding pockets in the PYR/PYL/RCARs which operate through a gate-latch-lock mechanism [97]. Taken together, these findings imply that ABA can interact with sites at the cytosolic region of the membrane and exert effects on the primary function of the protein, and this is different from the extracellular perception of ABA by the canonical receptor complexes PYR/PYL/RCAR [98], [99]. Since ABA can directly enhanced K^+^ efflux through GORK, it provides a direct, rapid, and alternative way to close the stomata in response to external stresses [95]. While the complete mechanism and biological role of this alternative ABA perception is yet to be fully understood, the findings have raised the possibility of additional direct ABA-interacting sites and ABA-responsive molecules that await characterization.

Using rationally designed variations of the ABA-binding motif, other candidate ABA-binding proteins have since been proposed and assessed structurally by modeling and molecular docking studies. Several were deemed as promising candidates for further experimental characterizations. For instance, the ABA motif DX{7,8}RX{3,4}DX{5,6}YX{6,7}H that resembles the latch part of plant ABA receptors [95], [97], [100], [101], identified 30 *Arabidopsis thaliana* proteins including GORK and these proteins are enriched in protein-binding related gene ontology (GO) terms whereas a less stringent version of the motif DX{7,8}RX{8,10}YX{6,7}H identified 182 proteins in the Arabidopsis proteome also with enrichments in the same GO terms and in addition to the terms “RNA polyadenylation” and “nitrogen compound metabolic process” [102]. ABA-binding sites in proteins downstream of ABA signaling pathway, or in proteins associated with ABA-dependent responses, were also identified [102]. This may point to a direct modulation of ABA of these proteins and/or involvement of ABA in processes mediated by them. Structural assessments of two representative human ABA-binding candidates: son of sevenless homolog 2, SOS2 (UniProt: Q07890) and exostosin-1, EXT1 (UniProt: Q16394) showed distinct cavities that could accommodate ABA and docking simulations also revealed favorable affinity for the ABA ligand. Two Arabidopsis ABA-binding candidates, serine/threonine-protein kinase, SRK2D/SnRK2.2 (TAIR: At3g50500) and serine/threonine-protein kinase, SRK2E/SnRK2.6 (TAIR: At4g33950) also yielded similar results [102].

We foresee that motif-based searches can also be applied to discover direct interactions between proteins and other plant hormones such as auxin, cytokinin, jasmonic acid, brassinosteroid, as well as peptide hormones. In the case of the Plant Natriuretic Peptide (PNP), a small peptidic hormone which functions as a systemic, extracellularly mobile regulator of plant metabolism and homeostasis as well as plant defense [103], [104], [105], [106], motif-based searches have already been successfully applied to identify several candidate molecules that interact specifically with PNP PNP-A (TAIR: At2g18660). The first interactor, the PNP-receptor 1 PNP-R1 (TAIR: At1g33612), was identified and isolated by affinity purification methods and motif searches have supported the discovery and guided the subsequent characterization of the intracellular GC domain in this receptor [107]. Other candidate PNP interactors have also since been experimentally validated. They include the *Arabidopsis thaliana* catalase 2 CAT2 (TAIR: At4g35090) and a Rubisco activase RCA (At2g39730), and in both cases, they harbor amino acid motifs similar to PNP-R1 that can be used to further guide the search PNP-protein interactions [108], [109], [110]. The ongoing discoveries of the growing PNP-interactome is one recent example that demonstrates the capability of motif searches in informing and supporting experimental elucidation of candidate ligands and/or interactors.

In another instance, it can be applied to the identification of auxin-binding sites beyond the TRANSPORT INHIBITOR RESPONSE 1/AUXIN-SIGNALING F-BOX (TIR1/AFB) auxin receptors. In recent years, a rapid and reversable auxin-dependent root responses has been identified [111], [112], [113]. Studies have shown that this response does not require transcriptional reprogramming afforded by the canonical auxin pathway. They are likely achieved through elevation of cytosolic Ca^2+^ and apoplastic alkalinization with a large part of the molecular mechanism underpinning the root rapid response to auxin remaining unresolved [114], [115]. Although this rapid response also involves the TIR1/AFB auxin receptors, the presence of additional auxin-binding sites in other downstream components such as ion channels, transmembrane receptors, or proteins directly involved in cytoskeletal organization, cannot be ruled out as these novel interacting sites would enable such versatility and explain the extraordinary fast dynamics observed in the root. The motif-based identification of ABA and PNP interaction sites as well as the many enzymatic sites (ACs/GCs) already characterized, have opened doors for the identification of similar hormone interaction sites in new proteins or invites a re-examination of existing ones. If indeed, ABA-binding sites are also identified in components of canonical and/or non-canonical auxin signaling pathways, it might explain the crosstalk between phytohormones and the dynamic regulation of their signal intensities. Several recent reports address antagonistic effects of ABA and auxin in primary root growth and ascorbic acid production in tomato where the novel and possibly common binding targets of ABA and auxin might cause this hitherto unresolved response signatures [116], [117].

## 3 Gas sensing

Heme containing proteins known as Heme-Nitric oxide/OXygen (H-NOX) contain highly conserved protein domains that bind to oxygen, carbon monoxide, and/or NO as ligands to affect important cellular and physiological processes [118], [119], [120], [121], [122], [123], [124], [125]. In obligate anaerobes, thermophilic bacteria, and nematodes, oxygen sensing H-NOX proteins provide a means for metabolic adaptation to prolonged hypoxia or to avoid oxygen-related reactions while NO sensing H-NOX proteins allow bacteria to regulate communal behaviors including biofilm formation [126], [127], [128], [129]. In animals including humans, H-NOX is the domain that binds to NO, thereby activating the GC to catalyze the conversion of GTP to cGMP, a second messenger that triggers vasodilation [130], [131]. As a signaling molecule that mediates many physiological processes in plants, NO has long been thought to carry out its function through post-translational modification of proteins such as S-nitrosation or S-nitrosylation [132], [133]. Canonical H-NOX proteins have not been identified through homology approaches in plants even though they are highly conserved in bacteria, fungi, animals, and other eukaryotes [125]. Extracting only the key amino acids at the ligand-binding site of H-NOX proteins, a search motif was created and applied to identify H-NOX moonlighting sites in plant proteins where the H-NOX motif HX{12,14}PX{14,16}YXSXR. This motif has identified a flavin monooxygenase AtNOGC1 (TAIR: At1g62580) as an NO sensing protein from *Arabidopsis thaliana* [134]. AtNOGC1 binds NO at a higher affinity than oxygen. A separate site on this protein was identified as a GC using a similar motif-based approach and experimental data has showed that the GC activity was enhanced through the binding of NO [134]. This is reminiscent of the NO-activated GC in other organisms including in humans [130]. AtNOGC1 was subsequently shown to participate in stomatal closure during the day through 8-nitro-cGMP which is induced by ABA and NO. Furthermore, signaling components such as Ca^2+^, cyclic adenosine-5′-diphosphate-ribose, and the SLOW ANION CHANNEL1 act downstream of the nitrated cGMP [135], thus linking NO perception to cGMP-dependent signaling much like in animal soluble GCs [130].

Another protein, an *Arabidopsis thaliana* DIACYLGLYCEROL KINASE 4 AtDGK4 (TAIR: At5g57690) was shown to harbor both GC and AC domains. The GC domain in particular, was discovered through a motif-based approach as well as predicted by GCPred, an online tool for the prediction motif-based GCs [136], [137]. Importantly, the same H-NOX motif identified AtDGK4 as a NO-sensing protein and *in vitro* characterization of AtDGK4 has showed NO-responsive spectral changes that are much reduced in AtDGK4 harboring mutations to key residues histidine (H) and tyrosine (Y) at the H-NOX moonlighting site [138]. Furthermore, Arabidopsis plants lacking DGK4, display slower pollen tube and NO-mediated growth and reorientation responses, implying desensitization to the NO signal. In the stigma, pollen tubes of these mutant plants were also outcompeted by the wild type as they were much shorter, and this resulted in poorer reproductive fitness [138]. It is tempting to draw parallels with the *Homo sapiens* male reproductive system that is also mediated by NO [139]. Unlike AtNOGC1, NO-sensing by AtDGK4 does not affect the GC activity thus implying different pathways governed by the two moonlighting sites of AtDGK4 which itself is an annotated lipid kinase [138]. A third H-NOX protein identified using the H-NOX motif is AtLRB3, which is a BTB/POZ domain-containing protein (TAIR: At4g01160) [140]. Annotated as responding to red light, AtLRB3, through NO perception, activates the photoreceptor phyB to promote red light-dependent photomorphogenesis through proteasomal degradation of growth-repressing transcription factors PIFs [141], [142]. Similarly, mutations to key residues in the H-NOX site markedly reduced the NO-dependent spectra [140].

These recent discoveries have opened a new direction of NO signaling research as novel candidates for NO-sensing hemoproteins have been identified using the original and derived H-NOX motifs such as HX{27,31}YXSXR [143], which has the proline (P) excluded since this residue was thought to be responsible for heme distortion and conferring oxygen sensing in obligate anaerobes, thus not crucial for NO-sensing [144]. Nearly 100 plant proteins were identified using this motif and the H-NOX candidates were subjected to bioinformatics analysis including GO-enrichments and co-expression analyses, revealing specific enriched and shared GO terms in catalysis, cation and nucleotide-binding, and transporter activity, including hydrolases and peptidases, as well as metals, transition metals and ATP-binding [143]. A novel prediction tool based on the H-NOX motif and properties of the intermediate amino acids HNOXPred, was created to enable rapid identification of potential H-NOX proteins across the kingdoms [145]. Importantly, this tool allows ranking of candidates based on how similar they are to the existing pool of H-NOX proteins identified by the motif. The majority of the candidates identified as “very probably H-NOX”, play important roles in the immune system and are implicated in cancers such as ADAMTS-16 which is upregulated by cancer-induced methylation in colorectal, lung, and oral cancers, a cervical cancer antigen interacting with high-risk HPV E6 proteins, the breast cancer antigen NY-BR38, and the 5T4 oncofetal antigen [146], [147], [148]. Other highly probable human H-NOX candidates participate in gene expression regulation and transport functions, while many bacterial H-NOX proteins are disease-causing bacteria, thus linking H-NOX-dependent NO sensing to diseases [145]. Besides offering mechanistic insights into cellular pathology, heme-based H-NOX gas sensors are becoming increasingly attractive not just as biological and chemical tools but also as drug targets for pharmacological use as well as for applications in cell biology and bio-catalysis [149].

## 4 Conclusion: Establishing interactomes and beyond

It is becoming increasingly clear that in cells, hundreds of different molecules are capable of forming specific but non-covalent interactions with each other and that the totality of these interactions constitute the cellular interactome [150], [151], [152], [153]. The protein surfaces are, after all, highly promiscuous with cavities and pockets that can accommodate many different biochemicals as part of their primary or moonlighting functions [154], [155], [156], [157], [158]. This feature combined with the structural malleability of proteins, present opportunities for specific interactions with localized signaling molecules and their interactions may determine the nature of downstream signaling events for example, serving as intersection points to switch from one pathway to another [38], [159], [160], [161], [162]. The interactome, much like the transcriptome, the proteome, and the metabolome, is dynamic and reflects developmental, spatial, and physiological response signatures [152], [153]. Interactions between molecules are due to specific affinities, often characterized by only short amino acid or nucleotide patterns that delineate the interacting sites which have in many cases been determined by mutation analyses [27], [32], [36], [42], [43]. Once such an interacting site has been experimentally confirmed, it can then serve as a starting point for the building of a motif that can assist in the discovery of other candidate proteins also interacting with the same ligand [5], [163]. Naturally, the more stringent these motifs are, the better the chance to obtain *bona fide* interacting sites [2]. It is reasonable to speculate that increasing insights into the role of the ever-growing number of specific interactions, peptidic or otherwise, will yield surprising new insights into the mechanisms governing complex cellular systems [6].

The application of carefully curated amino acid motifs to model and non-model organisms across the tree of life, therefore offers an exciting and currently undervalued opportunity for the discovery of hidden or unexpected ligand interacting sites in new and/or well-characterized proteins [107], [164]. This is in addition to the many examples of enzymatic moonlighting sites already identified [25], [27], [32], [36]. Discovering protein interactants beyond those involved in catalysis, could reveal novel signaling mechanisms or revisit existing ones [38], [107]. One such example is the recent identification of an AC operating in the TIR1/AFB auxin receptors which participate in the classical nuclear auxin signal transduction pathway that affects auxin-dependent root growth inhibition and gravitropism [164], [165]. A better understanding of interactomes is also likely to uncover complex hidden mechanisms that operate at a systems level [166], [167]. On the more applied side, insights form interactomes may address conceptual gaps in the literature and hence contribute to more effective and novel strategies for crop improvements and treatments of diseases among many other promising applications in cell biology, pharmacology, and bio-catalysis [149], [168], [169], [170], [171].

## Declaration of Competing Interest

The authors declare that they have no known competing financial interests or personal relationships that could have appeared to influence the work reported in this paper.
